# Supplementary material for: Defect-driven selective metal oxidation at atomic scale
Source: Nat Commun. 2021 Jan 25;12:558. doi: 10.1038/s41467-020-20876-9 (PMC7835350; doi:10.1038/s41467-020-20876-9)
Supplement: Supplementary file 1 — Supplementary Information [file 41467_2020_20876_MOESM1_ESM.pdf]

Supplementary Information for

Defect-driven Selective Metal Oxidation at Atomic Scale

Qi Zhu, Zhiliang Pan, Zhiyu Zhao, Guang Cao, Langli Luo, Chaolun Ni,
Hua Wei, Ze Zhang*, Frederic Sansoz*, Jiangwei Wang*

<u>This file includes:</u>

Supplementary Figures 1-15

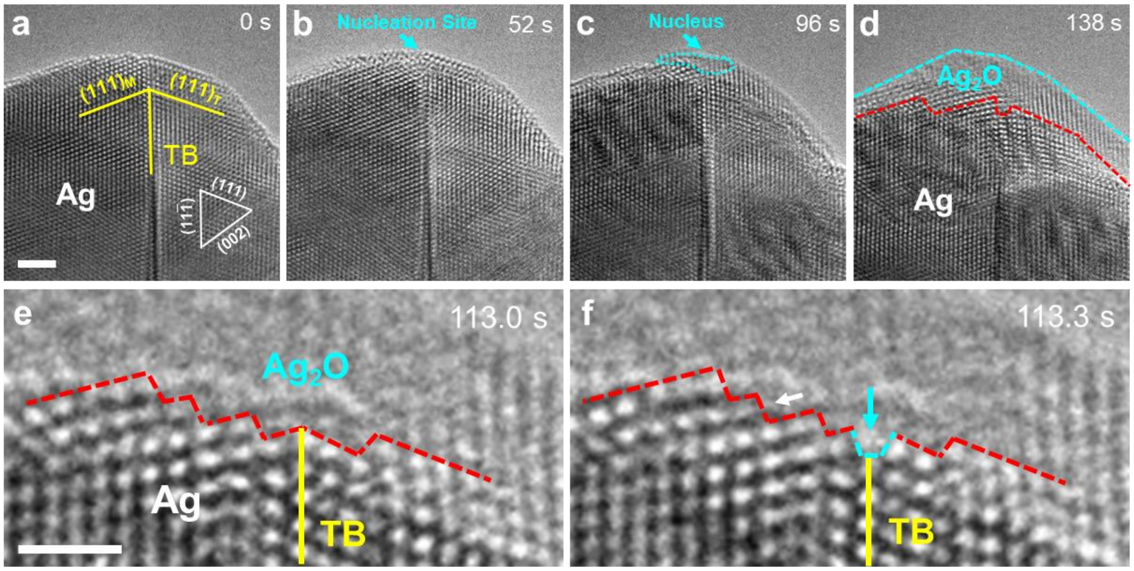

Supplementary Figure 1. Additional example showing the site-selective oxidation in a nanotwinned Ag nanocrystal. (a-d) Preferential oxide nucleation and growth at the TB-surface junction. The light blue arrows point to the preferential nucleation site of the Ag_2O embryo. (e-f) Zoomed-in images showing the preferential dissolution of atoms at TB-interface junction into Ag_2O and the migration of interface step during the oxide growth, as indicated by the light blue and white arrows, respectively. Scale bars: (a) 2 nm; (e) 1 nm.

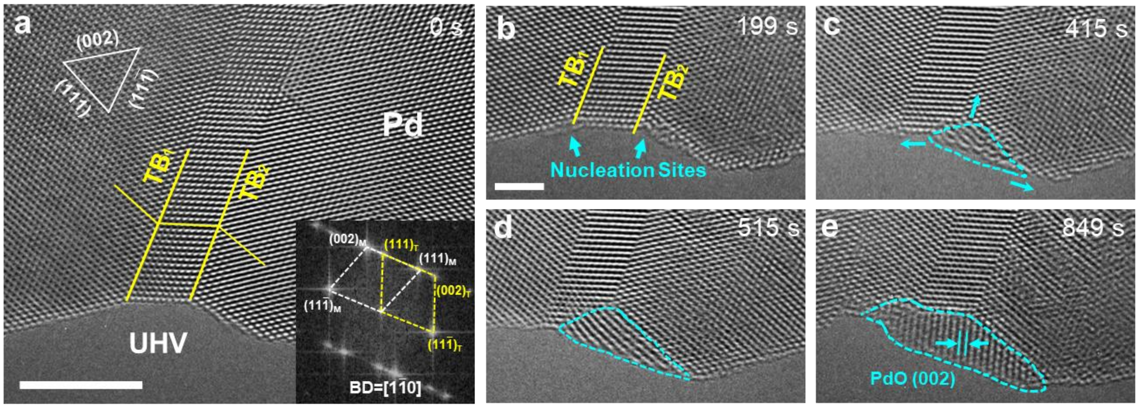

Supplementary Figure 2. Preferential oxidation at TBs in a Pd nanocrystal. (a) Pristine structure of the nanotwinned Pd. Inset is the FFT pattern of the nanotwinned Pd. (b-c) Preferential oxide nucleation at the TB-surface intersections (indicated by the light blue arrows). (d-e) Inward growth of the oxide (PdO) along the TB accompanied with lateral expansion along the surface. Scale bars: (a) 5 nm; (b) 2 nm.

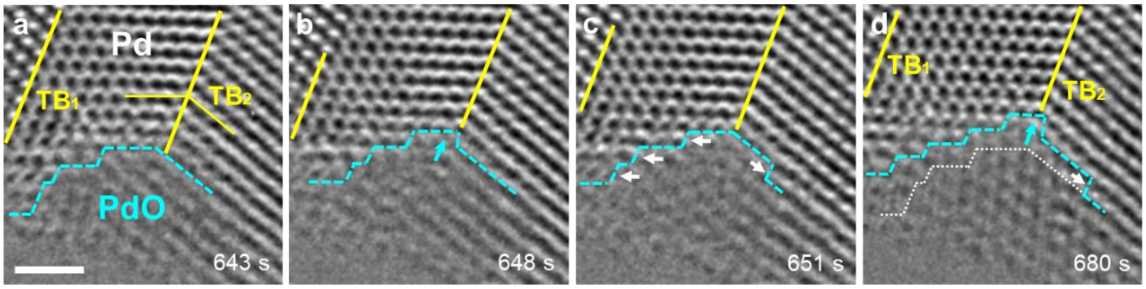

Supplementary Figure 3. TB-assisted layer-by-layer inward oxidation in a nanotwinned Pd nanocrystal. (a) Atomic structure of the Pd/PdO interface, which contains multiple steps. (b) Preferential oxidation of the atom column at TB-intreface junction that generates new steps (pointed out by the light blue arrows). (c-d) Layer-by-layer oxidation via the nucleation and migration of interface steps. The light bue dashed line and the white dotted line in (d) indicate the real-time and initial positions of the interface, respectively. Scale bar: 1 nm.

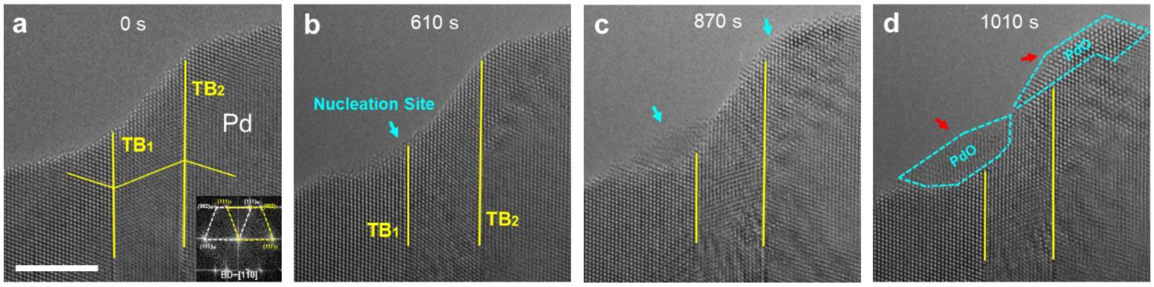

Supplementary Figure 4. Additional example showing the preferential oxidation at neighbouring TBs in a Pd nanocrystal. (a) A pristine Pd nanocrystal with two parallel TBs that intersect with the surface. Inset is the FFT pattern of the nanotwinned Pd. (b-d) Preferential oxidation at both TB-surface junctions. Scale bar: 5 nm.

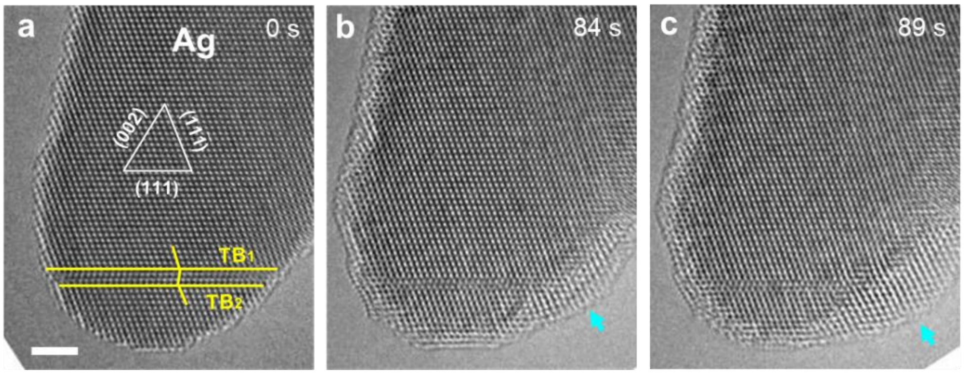

Supplementary Figure 5. Additional example showing the preferential oxidation at a transverse nanotwin away from the crystal tip. (a) Pristine Ag nanocrystal with an isolated transverse nanotwin. (b-c) Preferential oxide nucleation and growth at the TB-surface intersection site (indicated by the light blue arrow). Scale bar: 2 nm.

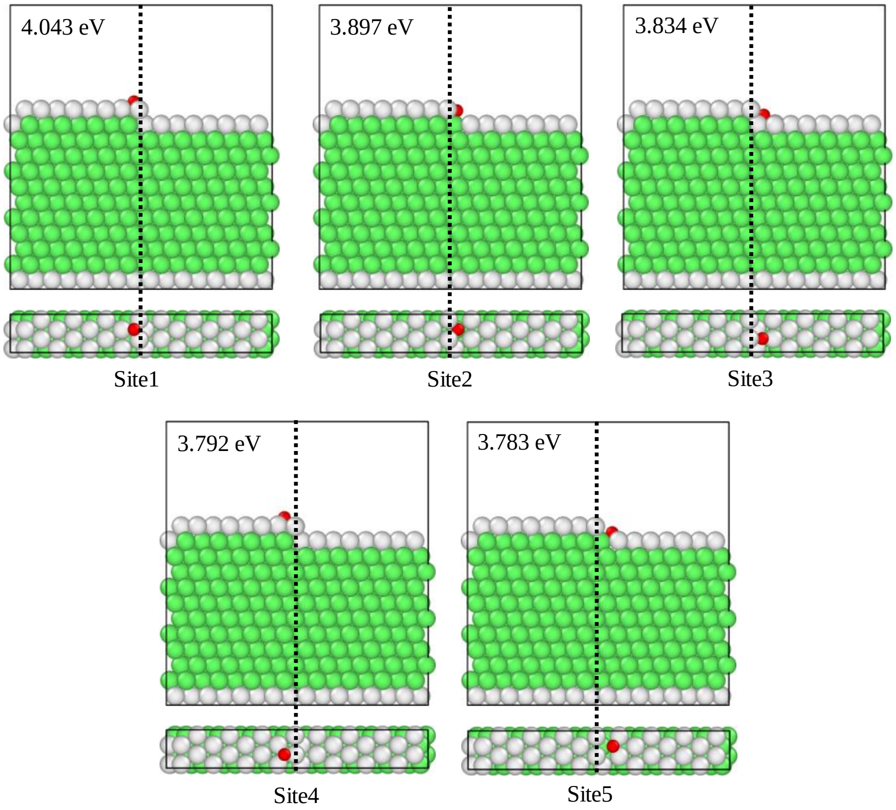

Supplementary Figure 6. Site-specific oxygen binding energy (E_b) near an atomic (111) surface kink step in a Ag single crystal with via density-functional-theory (DFT) calculation. The dotted reference lines indicate a kink step and the different adsorption sites of oxygen atom near this kink step are marked by the red dots.

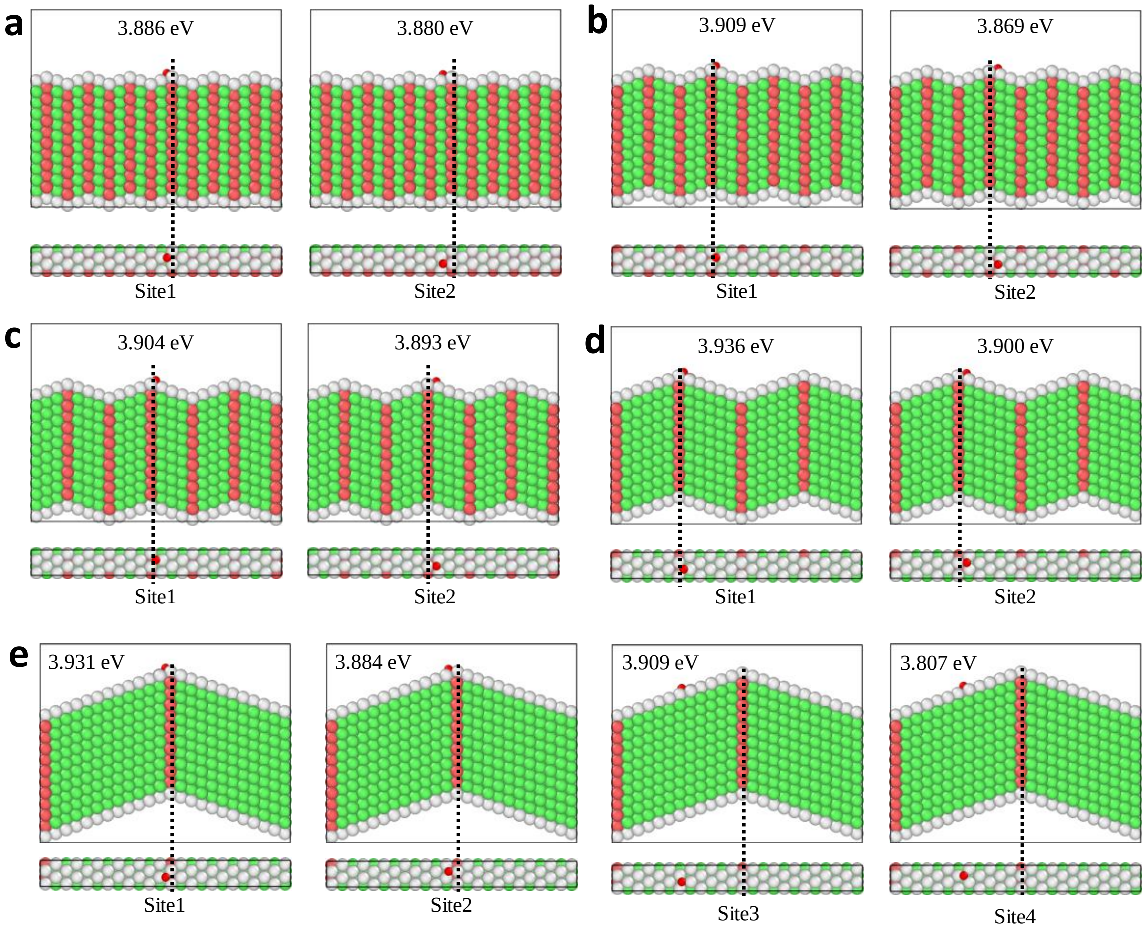

Supplementary Figure 7. Site-specific E_b of nanotwinned Ag nanocrystals with different TB spacings via DFT calculation. (a) Two (111) atomic layers (i.e., extrinsic SF configuration). (b) Three (111) atomic layers. (c) Four (111) atomic layers. (d) Six (111) atomic layers. (e) Twelve (111) atomic layers (isolated TB). The dotted reference lines indicate TB-surface junctions and the different adsorption sites of oxygen atom near the junctions are marked by the red dots.

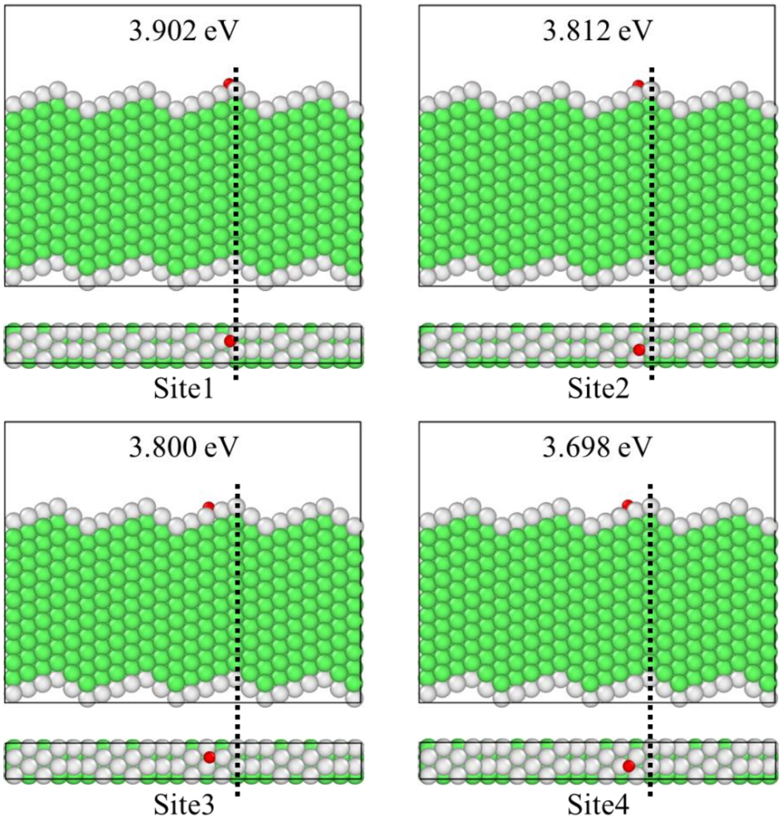

Supplementary Figure 8. DFT calculation of E_b at different adsorption sites in the Ag single crystal. The dotted reference lines indicate the junctions of surface facets and the different adsorption sites of oxygen atom near the junction on the surface are marked by the red dots.

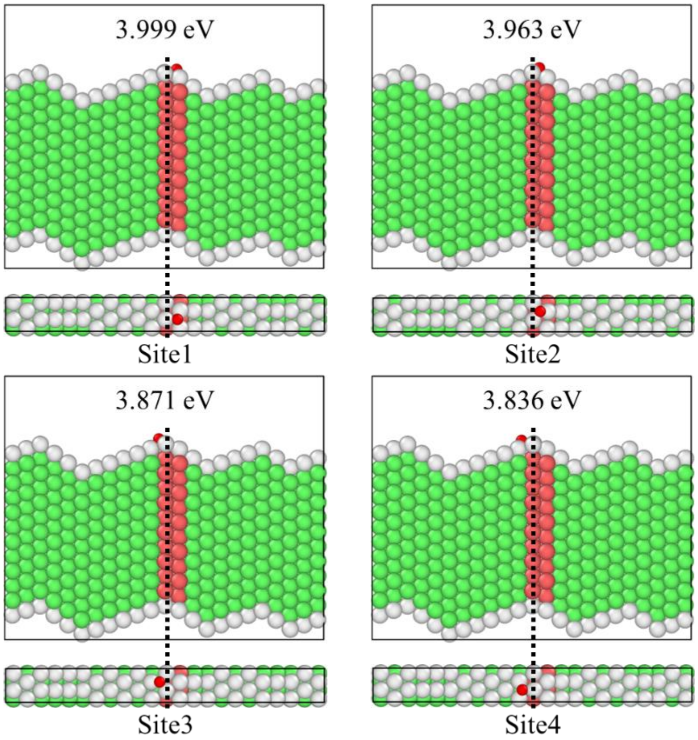

Supplementary Figure 9. Site-specific E_b at different surface adsorption sites near an isolated stacking fault (SF) via DFT calculation. The dotted reference lines indicate a SF-surface junction and the different adsorption sites of oxygen atom near this junction are marked by the red dots.

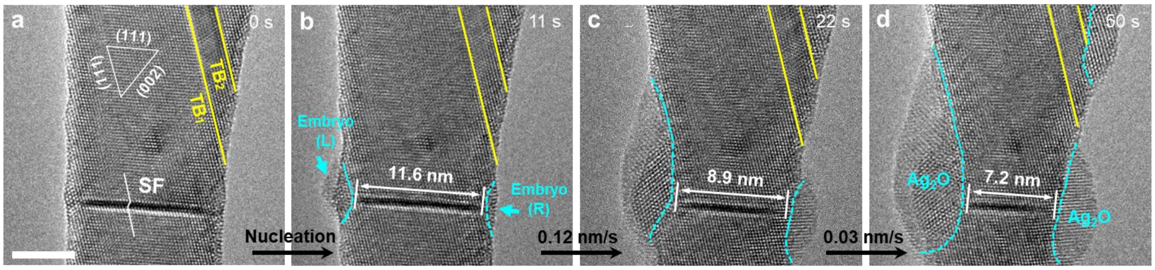

Supplementary Figure 10. Calculation of oxidation rate along the SF plane in Ag nanocrystal. (a) Pristine Ag nanocrystal with a transverse SF intesecting with both surfaces. (b) Simultaneous oxide nucleation at both SF-surface junctions. (c-d) Continuous inward oxide growth along the SF, where the distance between two oxide fronts (*L*) decreases from 11.6 nm to 7.2 nm. The average oxidation rate is determined by $-\Delta L/2\Delta t$, which gives 0.12 nm/s and 0.03 nm s^{-1} (with an average rate of 0.06 nm s^{-1}) for steady growth (b-c) and self-limiting (c-d) oxidation stages, respectively. Scale bar: 5 nm.

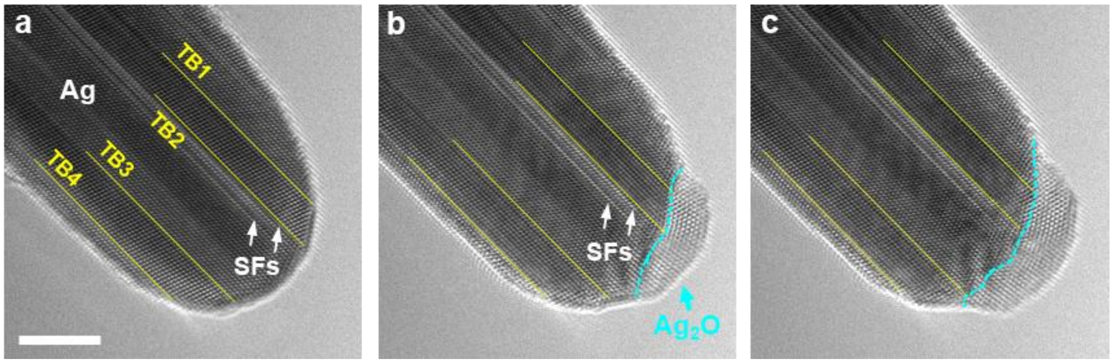

Supplementary Figure 11. Preferential oxidation in an Ag nanocrystal with multiple parallel twins and SFs. (a) Morphology of the pristine Ag nanocrystal. (b) Preferential oxide nucleation at the defect-surface intersection sites. (c) Subsequent inward and lateral growth of the oxide. Scale bar: 5 nm.

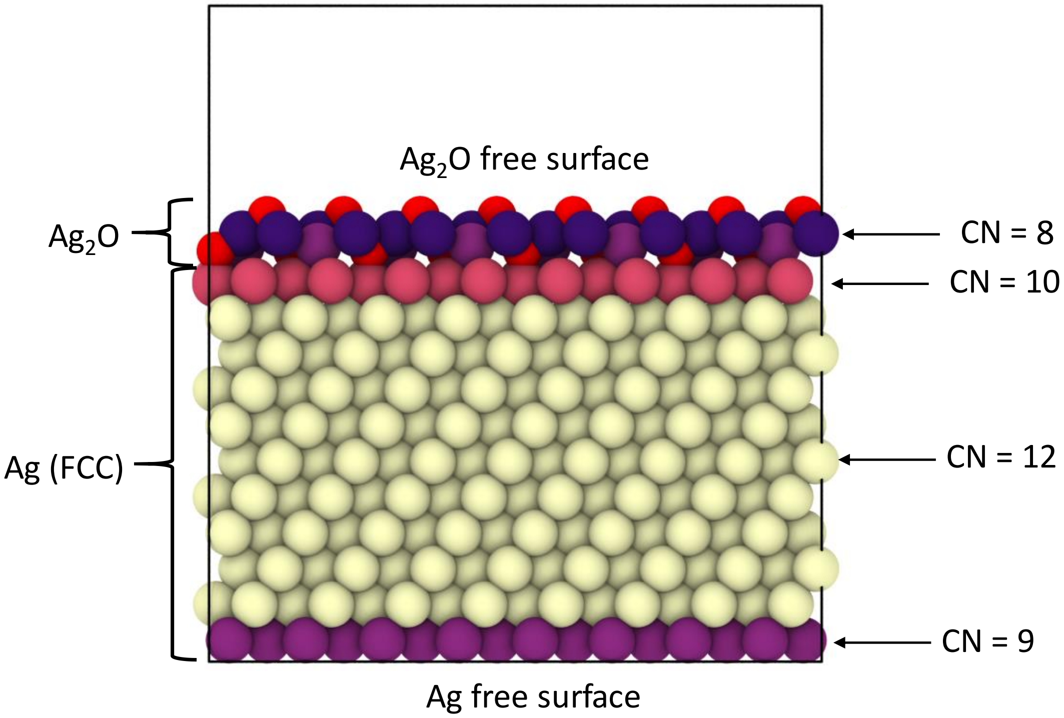

Supplementary Figure 12. Coordination numbers (CN) of atoms in a relaxed Ag-Ag_2O flat interface at equilibrium obtained by DFT calculation.

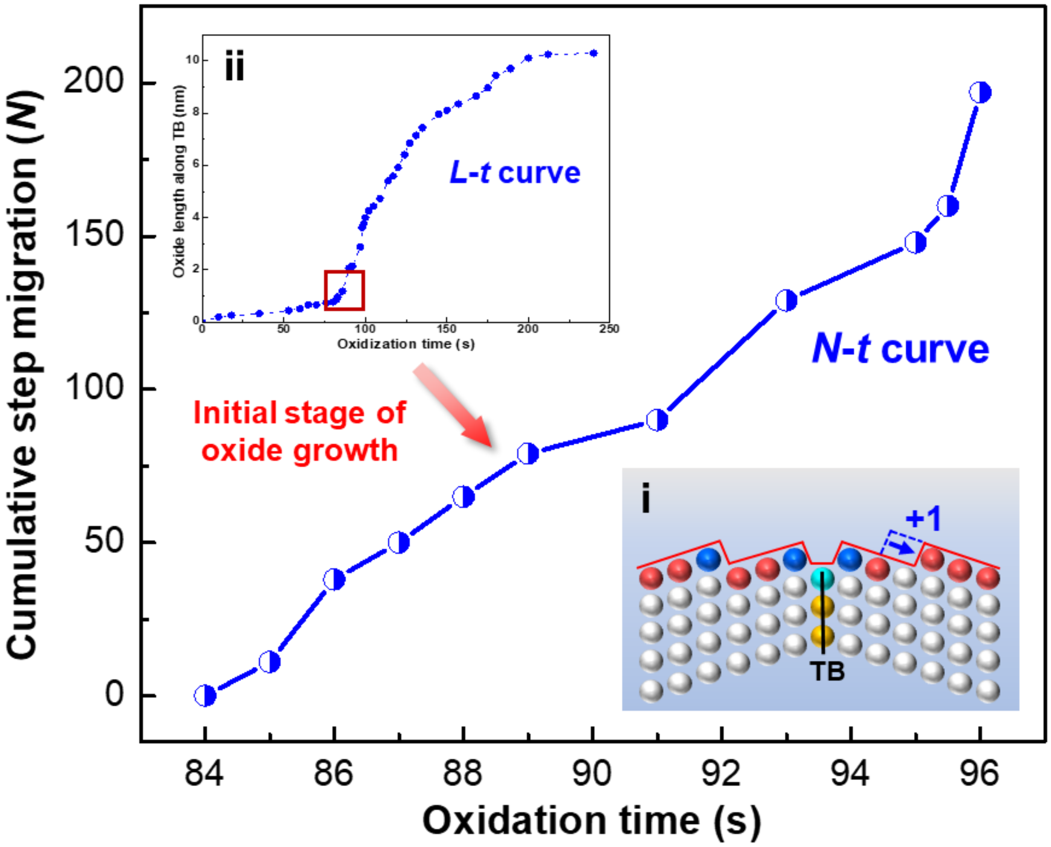

Supplementary Figure 13. Cumulative atomic step migration with increasing oxidation time (*N*-*t* curve) during the initial stage of oxide growth. The cumulative step migration *N* were recorded for all atomic steps along the metal-oxide interface with oxidation time (the track-and-count criterion for each step motion is schematically illustrated in inset i). Inset ii is the oxide thickness-time (*L*-*t*) plot in Fig. 4 of the main text, where the red square marks the initial stage of oxide growth.

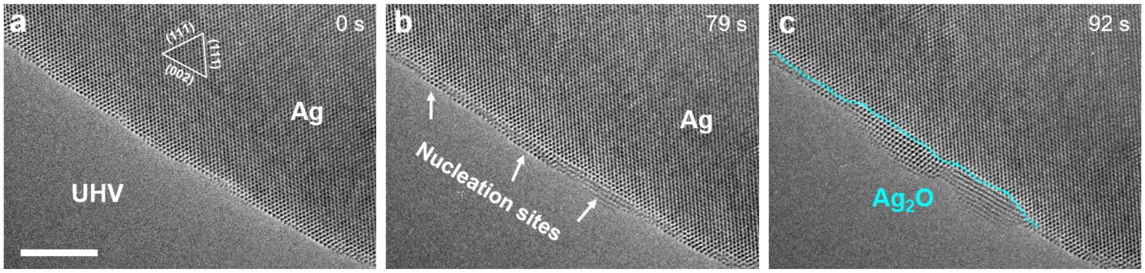

Supplementary Figure 14. Surface oxidation of a defect-free Ag single crystal. (a) Flat surface of the pristine Ag nanocrystal. (b) Homogeneous oxide nucleation at multiple sites on the surface. (c) Slow inward and lateral growth of the oxides. Scale bar: 5 nm.

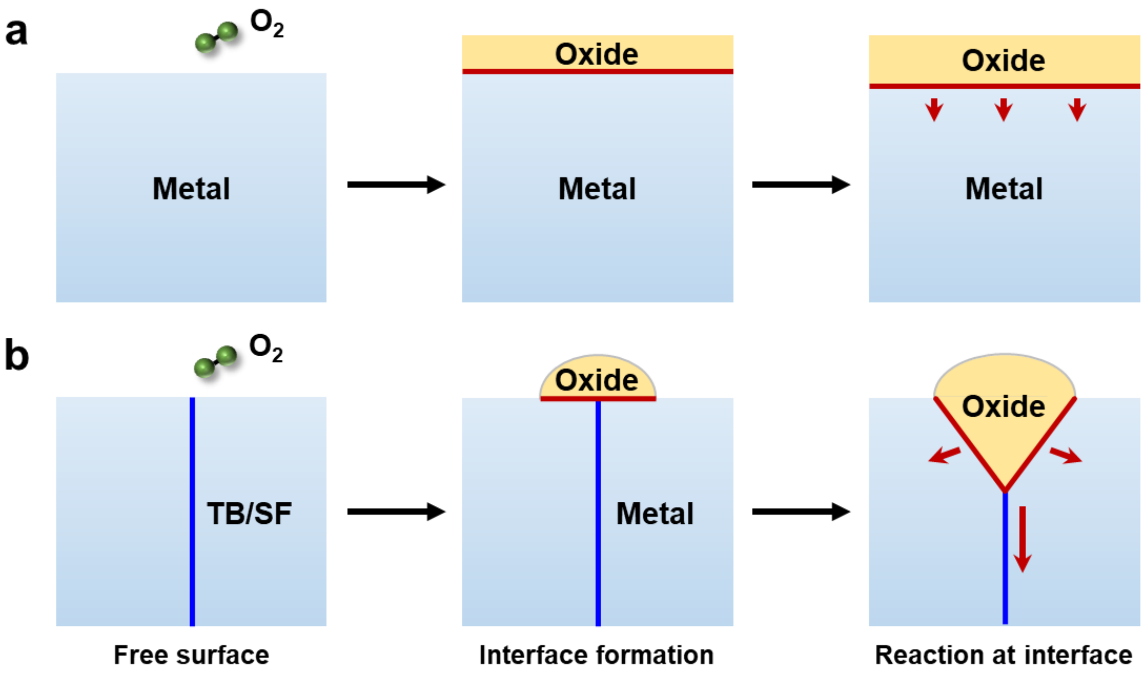

Supplementary Figure 15. Schematics showing the oxidation behaviours of metals. (a) Oxidation of metals without planar defects. (b) Oxiadation of metals with a planar defect intersecting the surface. The red lines delineate the oxide-metal interface and the length of the arrows schematically demonstrates the different migration rate of the metal/oxide interfaces.
